# The Precuneus Contributes to Embodied Scene Construction for Singing in an Opera

**DOI:** 10.3389/fnhum.2021.737742

**Published:** 2021-10-15

**Authors:** Shoji Tanaka, Eiji Kirino

**Affiliations:** ^1^Department of Information and Communication Sciences, Sophia University, Tokyo, Japan; ^2^Department of Psychiatry, Juntendo University School of Medicine, Tokyo, Japan; ^3^Juntendo Shizuoka Hospital, Shizuoka, Japan

**Keywords:** cognition, embodiment, imagery, mirror neurons, perspective, social, working memory

## Abstract

Performing an opera requires singers on stage to process mental imagery and theory of mind tasks in conjunction with singing and action control. Although it is conceivable that the precuneus, as a posterior hub of the default mode network, plays an important role in opera performance, how the precuneus contributes to opera performance has not been elucidated yet. In this study, we aimed to investigate the contribution of the precuneus to singing in an opera. Since the precuneus processes mental scenes, which are multimodal and integrative, we hypothesized that it is involved in opera performance by integrating multimodal information required for performing a character in an opera. We tested this hypothesis by analyzing the functional connectivity of the precuneus during imagined singing and rest. This study included 42 opera singers who underwent functional magnetic resonance imaging when performing “imagined operatic singing” with their eyes closed. During imagined singing, the precuneus showed increased functional connectivity with brain regions related to language, mirror neuron, socio-cognitive/emotional, and reward processing. Our findings suggest that, with the aid of its widespread connectivity, the precuneus and its network allow embodiment and multimodal integration of mental scenes. This information processing is necessary for imagined singing as well as performing an opera. We propose a novel role of the precuneus in opera performance.

## Introduction

Since the discovery of the default mode network (DMN), which is composed of the medial prefrontal cortex, precuneus, and angular gyrus (AG), it has been implicated in internal mentation, including mind wandering, self-projection, episodic memory processing, future thinking, and theory of mind (Buckner and Carroll, 2007; Szpunar et al., 2007; Fransson and Marrelec, 2008; Schacter et al., 2012; Raichle, 2015). While the DMN increases its activity during internal mentation, paying attention to external stimuli deactivates the DMN (Fox et al., 2005). This simplistic perspective is currently being developed. A functional magnetic resonance imaging (fMRI) study that used audio-visual movies reported pattern similarity in the DMN for movie viewing, listening, and recall (Zadbood et al., 2017). This suggests that the DMN is involved in encoding, reinstatement (spoken recall), and new construction of the same real-life episode. Furthermore, this study reported similarity between speakers’ and listeners’ neural patterns during movie viewing and mental construction, respectively, which was associated with increased comprehension of the described events in the listeners. The DMN is involved in naturalistic perception (Brandman et al., 2021). Therefore, the DMN mediates active and passive processing of mental imagery or scenery that can be shared with others.

There has been a recent increase in neuroimaging studies on the precuneus as a region with metabolic activity among the highest in the brain during the conscious resting state (Cavanna and Trimble, 2006). The precuneus is associated with various functions, including attention, spatial navigation, and mental imagery (Cavanna and Trimble, 2006; Sestieri et al., 2011; Freton et al., 2014). The precuneus is a relatively large cortical area located in the medial wall of the posterior parietal cortex (PPC; Cavanna and Trimble, 2006; Cauda et al., 2010). It possesses functional connectivity with numerous brain regions and is among the large network hubs in the brain (Margulies et al., 2009). Given its vicinity to the visual cortex (cuneus), the precuneus processes extensive visual information. However, several reciprocal connections with other cortical and subcortical areas indicate that mental imagery is multimodal and not limited to the visual modality. In conjunction with other network nodes, the precuneus can perform highly integrative processing. Accordingly, the precuneus mediates scene construction, which is defined as “the process of mentally generating and maintaining a complex and coherent scene or event” (Hassabis and Maguire, 2007). The precuneus, in conjunction with the posterior cingulate cortex, mediates complex scene processing; additionally, there are intercorrelations in the activity and metabolite levels of these regions during complex scene processing (Costigan et al., 2019).

The activation of the precuneus has also been observed in music processing (Cavanna and Trimble, 2006), which relies on mental imagery. It was previously reported that the functional connectivity of the precuneus in the resting condition was more extended and stronger in university students majoring in music than in those majoring in other disciplines (Tanaka and Kirino, 2016). The functional connectivity analysis of resting-state fMRI data revealed that compared with other students, music students had significantly higher connectivity of the precuneus with the opercular/insular regions, auditory areas in the superior temporal gyrus, and the lateral occipital cortex. A subsequent study reported enhanced multimodally associative connectivity in the thalamocortical network (Tanaka and Kirino, 2017). Moreover, a task-fMRI study that employed the imagined music performance paradigm reported increased functional connectivity within the DMN (e.g., connectivity between the precuneus and AG) as well as between the DMN and the hippocampus and amygdala during an imagined performance (Tanaka and Kirino, 2019). These findings lead to the question as to whether the precuneus is essentially involved in singing and performing an opera.

Opera usually involves onstage performance and comprises singing and acting by singers. Singers play roles in a scene as prescribed by the libretto, which refers to the text of the opera (Greenwald, 2014). Performing an opera requires character making, emotional expression, scene construction, and esthetic singing. Accordingly, multimodal scene construction in opera singers could be considered as a mental process involving the integration of numerous performance-related factors. Singers’ performance is dependent on such scenes mentally conceived by singers. Moreover, since the audience employs scene construction while appreciating an opera, singers and audiences could share mental scenes during an opera.

In a chapter of The Oxford Handbook of Music and Body, the authors state (p. 303): *“The living body of the singing actor on the operatic stage both has and performs a body, and the body is more than a biological entity. It is also an ideological construct*…. *The performer’s body is assigned the task by the director of presenting a role on stage in such a way as to ‘speak’ to the particular audience listening and watching”* (Hutcheon and Hutcheon, 2019). In opera performance, acting, which is required of singers, refers to bodily gestures that facilitate moving emotions in the audience (Williams, 2014). Although music directly accesses the characters’ emotions, acting facilitates the embodied emotion expression. Opera performance involves socio-emotional processing, where the performing singer should have empathy for the characters (Gallagher and Gallagher, 2020). Moreover, enacting a role requires the theory of mind, which modifies both singing and acting. Audiences do not read minds; rather, they infer minds through the audio-visual perception of the singing and acting. Performance imagery takes on embodiment, which influences the performance quality. A textbook for acting in opera states (p. 34): *“Salome is only one of many opera characters whose passions drive her to extreme measures. To perform her and her ilk effectively, you have to expand your own empathetic abilities and your willingness to explore and share the depths of the human souls”* (Ostwald, 2005). This performance could effectively stimulate embodied motor representation in the audience. The audience perceives the vivid performance of the singers by watching the actions of singers in an opera.

Scene construction could also activate the medial part of the prefrontal cortex (Hassabis and Maguire, 2009). Interestingly, a recent fMRI study showed that the ventromedial prefrontal cortex (vmPFC) was activated during autobiographical memory recall (McCormick et al., 2020). When performing a role of a character (a third person), however, the precuneus is activated and the activity of the vmPFC is rather suppressed, as demonstrated by an fMRI study of acting (Brown et al., 2019). Therefore, there seems to be a distinction between the vmPFC and precuneus in their contribution to scene construction including perspective-dependent information processing. In opera performance, the singer plays a third person, so that the precuneus is expected to play a central role in scene construction in opera performance.

This study aimed to investigate the contribution of the precuneus to singing in an opera. Since mental imagery processed in the precuneus is multimodal and highly integrative, we hypothesized that the precuneus is involved in opera performance by integrating multimodal information required for character performance in an opera. We tested this hypothesis by analyzing the functional connectivity of the precuneus during imagined singing and at rest. If the hypothesis is true, the precuneus will change its connectivity with other brain regions. Additionally, examining target brain regions of the reconfigured network can cause inference to the contribution of the precuneus to mental scene construction and intracerebral processing for opera performance.

## Materials and Methods

### Ethical Issues

All study procedures were approved by the ethics committees of Sophia University and Juntendo University, Japan. This study conformed to the tenets of the Declaration of Helsinki. All participants provided written informed consent before participating in the study.

### Participants

All the participants were vocalists (*n* = 42, females 37, age 19–44 years, and mean age: 27.0 years) who had experience of performing opera on stage. The participants were Japanese and healthy, without a history of neurological or neuropsychiatric diseases.

### Task

All participants completed two sessions, namely the imagined music performance session and the resting-state session. During the imagined music performance session, participants were asked to vividly imagine the act of singing in an opera on stage, with their eyes closed and without performing actual movements. The “performed” music was freely chosen from their repertoires (e.g., “Regnava nel silenzio” from *Lucia di Lammermoor* by G. Donizetti and “Da, chas Nastal! Prostite Vy” from *The Maid of Orleans* by P.I. Tchaikovsky). The performance was truncated at the end of each session. In the resting-state session, the participants were asked to close their eyes and not to think about anything specific.

### Image Acquisition

We acquired whole-brain images using a Philips Achieva 3.0-T MRI scanner equipped with a 32-channel head coil array. We collected high-resolution T1-weighted images for anatomical reference, using a 3D magnetization-prepared rapid acquisition gradient echo sequence: echo time (TE) = 3.3 ms, repetition time (TR) = 15 ms, flip angle = 10°, matrix size = 180 × 256 × 256, and voxel size = 1 mm × 1 mm × 1 mm. The total image acquisition time was 3 min and 31 s.

We collected blood oxygen level–dependent (BOLD) fMRI data during imagined music performance and resting-state sessions. A T2^∗^-weighted gradient-echo-planar imaging sequence was used with the following parameters: TE = 30 ms, TR = 2,000 ms, flip angle = 90°, field of view = 240 mm × 240 mm, matrix size = 64 × 64, number of axial slices = 33, and voxel size = 3.75 mm × 3.75 mm × 4.00 mm. Slices were acquired in the interleaved ascending order, starting with odd-numbered slices and followed by the even-numbered slices. Each session comprised 200 scans. The image acquisition time was 6 min and 40 s.

### Preprocessing

We preprocessed imaging data using the CONN toolbox v.20.b (Whitfield-Gabrieli and Nieto-Castanon, 2012), in conjunction with Statistical Parametric Mapping, version 12 (Wellcome Department of Cognitive Neurology, London, United Kingdom; http://www.fil.ion.ucl.ac.uk/spm), running on MATLAB version R2021a (MathWorks, Inc.). Individual fMRI data were co-registered with the T1 images. The fMRI data were realigned, slice-timing corrected, and normalized to the standard Montreal Neurological Institute template, as implemented in the Statistical Parametric Mapping software platform. We processed image artifacts originating from head movement using the ART-based scrubbing procedure as an artifact removal tool (Nieto-Castanon, 2020). Signal contributions from the white matter, cerebrospinal fluid, and micro-head movements (six parameters) were regressed out of the data. Finally, the fMRI data were band-pass filtered (0.008–0.09 Hz) and functional images were spatially smoothed using a Gaussian filter kernel (full width at half-maximum = 8 mm) for subsequent seed-to-voxel analysis.

### Analyses

The CONN toolbox was used for seed-to-voxel functional connectivity analysis, with the precuneus as the seed. We calculated Pearson’s correlation coefficients between the time course of the precuneus and those of all other voxels in the gray matter, which yielded a seed-to-voxel connectivity matrix. Positive and negative correlation coefficients defined positive and negative functional connectivity, respectively, (Whitfield-Gabrieli and Nieto-Castanon, 2012). Subsequently, the correlation coefficients were converted into normally distributed scores using Fisher’s transformation and used in the population-level analysis. Within the same sample, we statistically analyzed between-condition differences in functional connectivity (imagined music performance and resting). We applied a height threshold of *p* < 0.001 (uncorrected) to individual voxels for defining clusters. Subsequently, the significance level for the extracted clusters was set to *p* < 0.05, with family-wise error (FWE) correction.

For visualizing and analyzing the network reconfiguration of the precuneus during the task, we also performed the region of interest (ROI)-to-ROI analysis of functional connectivity using the CONN toolbox. The ROI set implemented in the CONN toolbox was based on the Harvard–Oxford atlas, which contains 132 ROIs covering the whole brain. For each participant, we calculated the mean BOLD time course within each ROI; further, the correlation coefficients were calculated from the time courses. The correlation coefficients were converted into normally distributed scores using Fisher’s transformation. Moreover, we calculated the connectivity matrix of the precuneus with the remaining ROIs. Within the same sample, we statistically tested between-condition differences in functional connectivity. The threshold for between-condition differences in the connectivity matrix was set at *p* < 0.05, with false discovery rate (FDR) correction.

## Results

**FIGURE 1:**
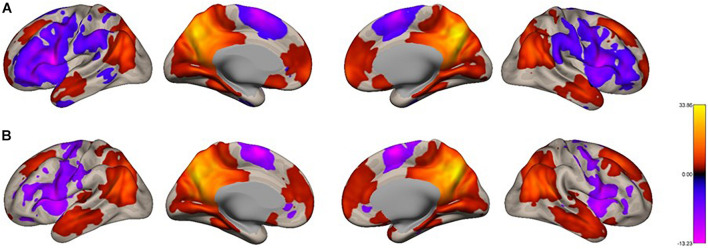
Voxel-level functional connectivity maps of the precuneus as the seed. **(A)** resting, **(B)** imagined singing. We applied a height threshold of *p* < 0.001 (uncorrected) to individual voxels for defining clusters. Subsequently, the significance level for the extracted clusters was set to *p*-FWE < 0.05.

**FIGURE 2:**
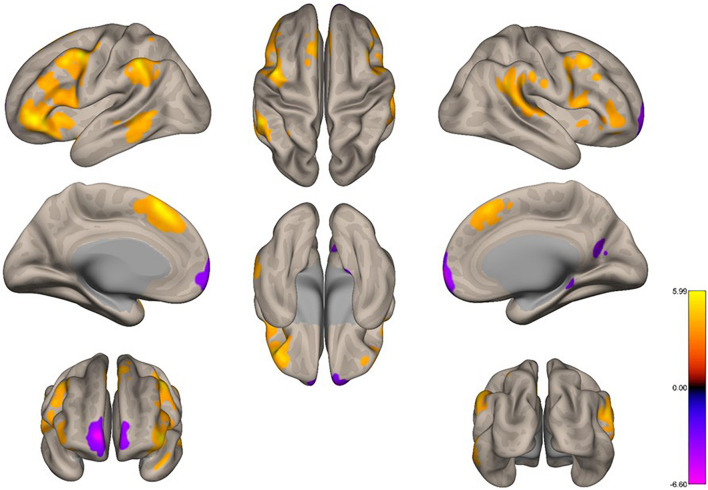
Voxel-level changes in the functional connectivity of the precuneus between the task condition and the resting condition. We applied a height threshold of *p* < 0.001 (uncorrected) to individual voxels for defining clusters. Subsequently, the significance level for the extracted clusters was set to *p*-FWE < 0.05.

We analyzed the functional connectivity of the precuneus during imagined music performance and resting condition, yielding seed-to-voxel functional connectivity maps with the precuneus as a seed. In both conditions, the precuneus had positive connections within the DMN (Figure 1). Furthermore, regions in the temporal cortex were connected to the precuneus. Notably, in the resting condition, large areas in the PFC (both lateral and medial), as well as part of the parietal and posterior temporal region, showed negative functional connectivity with the precuneus. Figure 2 shows the between-condition differences in connectivity. During the performance task, the precuneus showed increased functional connectivity with the lateral middle frontal gyrus (MFG), inferior frontal gyrus (IFG), medial side of the superior frontal gyrus (SFG), and portions of the parietal and temporal cortices at statistically significant levels. Small parts of the anterior and medial posterior cortices showed decreased connectivity during the task, which was also statistically significant.

**FIGURE 3:**
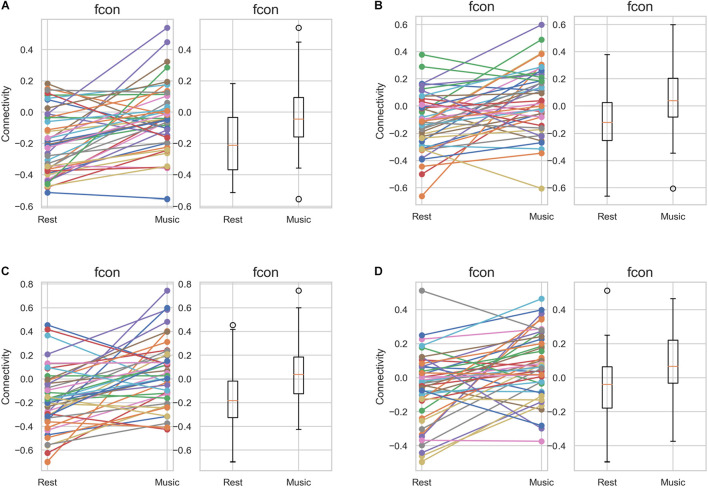
Between-group comparison of the functional connectivity of the precuneus in the individual level. The results of all the 42 participants are depicted, distinguished by different colors. **(A)** left OFC (*t* = 4.65, *p* = 0.000034), **(B)** left MFG (*t* = 3.97, *p* = 0.00028), **(C)** left pSMG (*t* = 4.39, *p* = 0.000077), and **(D)** left SFG (*t* = 3.88, *p* = 0.00037). MFG, middle frontal gyrus; OFC, orbitofrontal cortex; pSMG, posterior supramarginal gyrus; and SFG, superior frontal gyrus.

**FIGURE 4:**
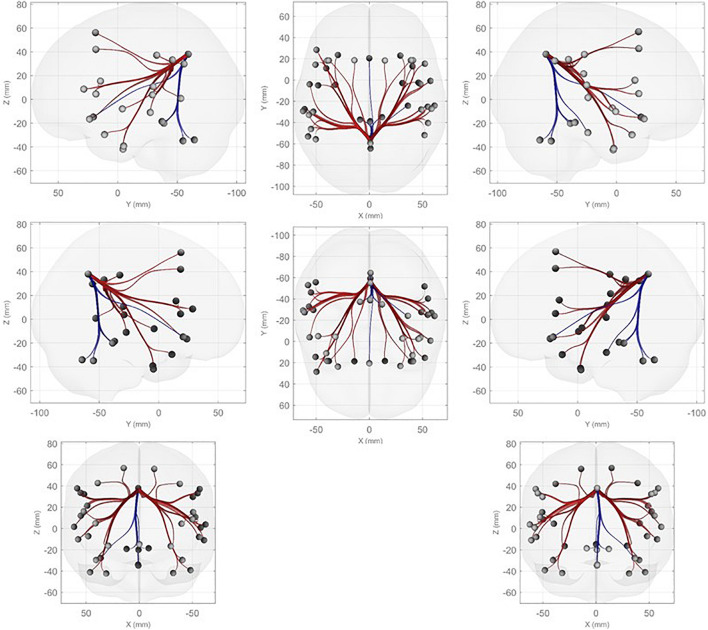
ROI-to-ROI functional connectivity analysis with the precuneus as the seed. Connectivity that was increased (decreased) during the performance condition compared with the resting condition is indicated in red (blue) based on the criteria of *p*-FDR < 0.05.

**FIGURE 5:**
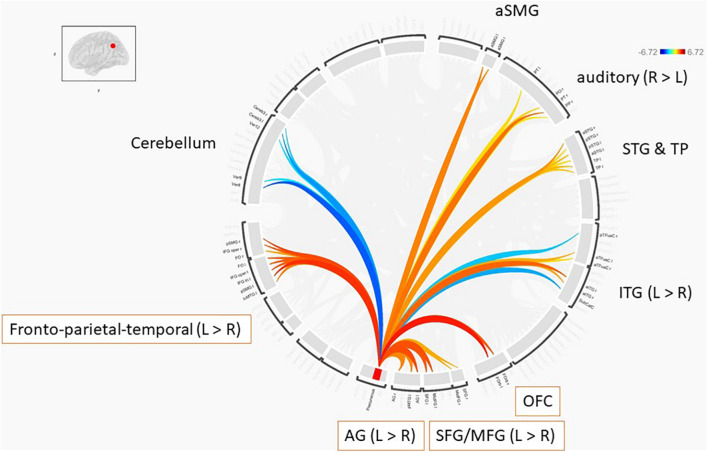
Clustered ROI-to-ROI functional connections with significant between-condition differences based on the criteria of *p*-FDR < 0.05.

Further, we performed ROI-to-ROI functional connectivity analysis. Generally, there were larger functional connectivity changes of the precuneus in the left than in the right hemisphere. Figure 3 shows functional connectivity changes between the precuneus and left orbitofrontal cortex (OFC; A), left MFG (B), left posterior supramarginal gyrus (SMG) (C), and left SFG (D). Further, we performed between-condition comparisons; Figure 4 shows a visualization of the connectivity changes between the two conditions. The precuneus showed increased connectivity with ROI sets in the frontal, temporal, and parietal cortices as well as decreased connectivity with ROIs in the cerebellum and posterior-inferior temporal cortex during the performance task than during the resting condition at statistically significant levels. The functional connections with significant between-condition differences were clustered, as seen in Figure 5. Marked increases were observed in the fronto-parieto-temporal, AG, SFG/MFG, and OFC clusters, while a marked decrease was observed in the cerebellum cluster.

## Discussion

Our findings revealed increased functional connectivity of the precuneus with several brain regions during imagined singing compared with the resting condition at statistically significant levels. These brain regions included the lateral part of the prefrontal and parietal cortices, posterior temporal cortex, and dorsomedial prefrontal cortex (dmPFC). These regions overlap with several functional networks, including the language, working memory, mirror neuron, and theory of mind networks, indicating that imagined singing requires highly integrated across-domain information processing. Our findings are discussed in the subsequent sections.

### Extended Language Network

We observed leftward asymmetry of regions showing increased connectivity with the precuneus, including the IFG, SMG, and inferior temporal gyrus, which are nodes of the perisylvian language network (Catani et al., 2005; Powell et al., 2006; Xiang et al., 2010; Friederici, 2011; Hagoort, 2013). The increased functional connectivity with the IFG suggests that imagined singing utilized the language production system. Previous studies have shown that the language network is connected with other brain regions related to general cognitive functions (Hagoort, 2013, 2017; Hertrich et al., 2020). Communication, which involves language processing as well as speech production and comprehension, is accomplished by extended networks, the language core and its beyond. The extended language network is not restricted to the left hemisphere. The right hemisphere is also activated in subprocesses, such as metaphor comprehension (Ferstl et al., 2008). Although not exclusive, increased connectivity with the perisylvian regions in the right hemisphere suggests affective prosody (Sammler et al., 2015; Hertrich et al., 2020), which is important for singing. Note that there was also an increase in the connectivity with the superior temporal gyrus, including the planum temporale, in the right hemisphere. An fMRI study showed that, comparing singing with speech, singing activated the planum temporale in the right hemisphere more than speech (Callan et al., 2006). This region and its anterior adjacent region, the auditory cortex, process sound imagery, as suggested by other fMRI studies (Bunzeck et al., 2005; Regev et al., 2021).

The inferior parietal lobule (IPL), specifically the SMG, is involved in several aspects of speech and language control, including speech learning, sensorimotor adaptation, phonological processing, and speech monitoring (Hickok and Poeppel, 2007; Hartwigsen et al., 2010; Shum et al., 2011; Kort et al., 2014; Simonyan and Fuertinger, 2015). Therefore, our findings suggest that the SMG contributes to singing. Furthermore, we found increased connectivity between the precuneus and right SMG, which suggests that melody, similar to prosody in speech, was integrated into speech processing. In these processes, lyrics and emotions are integrated into the mental scenes of singers. Taken together, our results lead to the notion that an increase in functional connectivity between the precuneus and extended language network contributes to the generation of mental scenes by integrating language with emotional expression for singing.

### Frontoparietal Network

The inclusion of the frontal and parietal regions in our result suggests the involvement of *working memory* in imagined singing. The network for working memory extends over a wide brain region, which not only includes the lateral prefrontal cortex (LPFC) and PPC as core regions, but also sensory regions, such as visual, auditory, and somatosensory cortices (Christophel et al., 2017). This wide network distribution reflects the nature of working memory, which represents sensory information and behavioral plans at an abstract level. Our findings indicated that regions exhibiting increased connectivity with the precuneus included the frontal, parietal, temporal, and auditory regions. The lack of involvement of the other sensory regions could be attributed to the task of “imagined singing” involving keeping the eyes closed and the body still. This result is consistent with the feature that imagined singing is a memory-driven, rather than sensory-dependent, task.

### Mirror Neuron Network

Notably, we observed increased functional connectivity of the precuneus with the mirror neuron network, which is composed of the IFG, IPL, and pSTS/pMTG (Iacoboni and Dapretto, 2006). Since mirror neuron activity is related to actions, imagined actions could evoke mirror neuron activity in the participants. The increase of connectivity between the precuneus and the mirror neuron network suggests that the mental scene is *embodied*. A recent electroencephalography study detected mirror neuron activity during audiovisual appreciation of an opera (Tanaka, 2021). However, only listening to singing did not induce mirror neuron activity. Therefore, it is rational to interpret that mirror neuron activity was elicited by integration with the visual perception of a singer’s action rather than the sounds themselves. Embodiment is required in opera performance to allow the audience to perceive vivid imagery of the opera. The embodied cognition approach proposes a new perspective regarding the format of mental imagery (Palmiero et al., 2019), and our findings are compatible with this perspective. The cooperation between the DMN and the mirror neuron network could yield the appropriate processing required for social-cognitive demands (Molnar-Szakacs and Uddin, 2013). Therefore, embodied representation involving the mentalization of the character’s mind is characterized by increased functional connectivity between the precuneus and the mirror neuron network. This notion is consistent with recent findings that the precuneus was activated significantly higher when describing a person and activity than when describing a place and object in an autobiographical memory recall task (Gilmore et al., 2021). Ostwald, who is a director of operas and plays, wrote the following in his book (p. 12): *“Imagine that you are so deeply involved that your feelings cause you to utter sounds. When you immerse yourself in your character’s circumstances so that what he [the character] must sing and do seems not just logical but inevitable, you can let your outward body movements spring from your own internal impulses and feelings. Working this way, you will choose evocative and believable actions unconsciously, but intuition”* (Ostwald, 2005).

A recent study demonstrated the involvement of the DMN in imagined music performance (Tanaka and Kirino, 2019). By embracing imagery in their minds, singers perform by enacting their roles in the opera. In their brains, integration of various information types is necessary for expression in performance. This is due to the demands of singers to make characters in a scene, which have numerous parameters in the scene. Finding the parameters and unifying gestures while incorporating one’s personality ultimately shapes the character (Clark, 2002). Additionally, singers are required to empathize with their characters (termed as “empathic embodiment”), which is aided by conceiving mental imagery of the characters’ emotional states (Heisel, 2015).

### Temporoparietal Junction

In the right hemisphere, there was increased connectivity between the temporoparietal junction (TPJ) and the precuneus during imaging singing. The TPJ is the major site of social cognition, including the theory of mind (Kubit and Jack, 2013; Schurz et al., 2014). The theory of mind refers to the “ability to explain and predict other people’s behavior by attributing to them independent mental states, such as beliefs and desires” (Gallagher and Frith, 2003). When singing in an opera, the singer performs as the character being played, which requires the inference of the mental state of the character in a certain situation. In dramas, actors play a role. A recent study reported that the precuneus is more strongly activated when actors played other characters than when they were themselves (Brown et al., 2019). Human animacy could activate the precuneus and theory of mind network (Cross et al., 2016). The right TPJ is associated with embodied processes relevant to perspective taking (Gallese, 2007; Seymour et al., 2018; Martin et al., 2020). Therefore, both the precuneus and TPJ could be involved when acting a character in an opera. This study suggests that scene construction in imagined singing involves *theory of mind*.

### Dorsomedial Prefrontal Cortex

During imagined singing, there was increased functional connectivity between the precuneus and dmPFC, which is associated with social interaction (Wagner et al., 2016) and emotion regulation (Wager et al., 2008). To assess whether the dmPFC is specialized for social information processing, a previous fMRI study analyzed the response in this brain region when natural viewing of a movie scene. It reported a strong tuning of this region to the social features of this stimulus. Notably, the dmPFC was the only region with a significant correlation between its activity and the agreeableness trait during a theory of mind task (Arbula et al., 2021). The agreeableness trait is among the five personality dimensions (McCrae and John, 1992), with participants having this personality trait being empathetic. Another fMRI study reported that the dmPFC and other cortical midline structures, including the vmPFC, precuneus, and PPC, were activated more strongly when viewing others’ actions through affective social interactions than through neutral cooperation (Arioli et al., 2018). Therefore, our findings suggest that the mental scenes constructed in our participants included the *socio-emotional* factors required for performance.

### Orbitofrontal Cortex

Increased functional connectivity of the precuneus with OFC was higher than that with any other region (left hemisphere: *t* = 4.65, *p* = 0.000034; right hemisphere: *t* = 3.86, *p* = 0.00039). This finding was unexpected as we did not suppose the involvement of the OFC in scene construction. The OFC is associated with reward processing and decision making, which is required for complex and flexible emotional and social behavior (Elliott et al., 2000; Kringelbach and Rolls, 2004; Suzuki et al., 2017; Lopez-Persem et al., 2020). There is functional segregation in the OFC. Specifically, the lateral and medial sections mediate reward processing and decision making, respectively, (Noonan et al., 2012). We infer that the reward system is involved in the construction of mental scenes during imagined singing by recollecting pleasure episodic memories previously experienced by the participants on stage.

### Network Interaction

Our findings revealed a novel pattern of network reconfiguration or interaction with other networks during imagined music performance, which is a naturalistic condition. The DMN was originally characterized under resting conditions. Execution of numerous experimental tasks has been shown to deactivate the DMN, which was subsequently considered task-negative (Fox et al., 2005). Functional connectivity analysis of resting-state fMRI data revealed inhibitory functional connectivity of the DMN with executive networks, including the frontoparietal network (Murphy et al., 2009). Distinct network groups appeared to be task-positive and task-negative (Jack et al., 2013). However, this dichotomy was found to be more nuanced than previously assumed (Piccoli et al., 2015). Social cognitive tasks activate brain regions overlapping with the DMN (Schilbach et al., 2006; Mars et al., 2012); moreover, goal-directed cognitive tasks co-activate the DMN and executive networks (Spreng et al., 2010; Meyer et al., 2012). A recent graph theoretical analysis suggested that the DMN cooperates with, rather than inhibits, the frontoparietal network and language network (Gordon et al., 2020). This inter-network connectivity allows these networks to perform highly integrated information processing. In complex situations, the brain mediates multiple-domain processing through dynamic network reconfiguration (Mineroff et al., 2018; Wen et al., 2020). The DMN could be involved in cognitive task execution (Vatansever et al., 2015a). Here, the DMN and working memory network are not anti-correlated during all phases of working memory processing (Piccoli et al., 2015). Moreover, the DMN could be involved in socio-affective processing (Amft et al., 2015) and global functional integration (Vatansever et al., 2015b). The notion that the DMN is involved in imagined singing is consistent with these findings. Our study demonstrated another situation where the DMN, particularly the precuneus, cooperates with executive networks.

### Disinhibition

In our study, resting-state negative/inhibitory functional connectivity of the precuneus tended to weaken or become positive during task performance, with numerous studies reporting this feature of connectivity changes. For example, functional connectivity between the DMN and working memory network changes in different phases of the working memory task (Piccoli et al., 2015). Moreover, the negative connectivity between the LPFC and posterior cingulate cortex/retrosplenial cortex in the maintenance phase is nullified in the encoding and retrieval phases, whereas the connectivity between the left LPFC and IPL became positive. Recently, we observed a similar phenomenon of dynamic changes in functional connectivity in patients with fibromyalgia (Usui et al., 2020). The patients listened to a piece of classical music, “Duo for Violin and Viola in G Major, K.423” by W. A. Mozart, with fMRI being performed before and after the session. The pain measure was significantly reduced by listening to the music. Simultaneously, reciprocal inhibition between the precuneus and insula was significantly reduced. The insula has been reported to mediate pain, sensory, motor, and emotional processing (Cauda et al., 2011).

### Limitations

Although there was increased functional connectivity of the precuneus with many regions during the imagined singing task, few regions showed decreased connectivity, including the cerebellum, inferior temporal gyrus and portions of the anterior prefrontal cortex. For example, the cerebellum has functional connectivity with the DMN, attentional, executive, language, and salience networks in the resting state (Habas, 2021). Therefore, the decreased connectivity of the cerebellum might be involved in performance processing. Alternatively, it may be indicative of behavioral inhibition, which was required to the participants. Further studies are needed to interpret or examine these results.

## Conclusion

Our findings demonstrated that, during imagined singing, there were statistically significant levels of increased functional connectivity between the precuneus and brain regions related to language, mirror neuron, socio-cognitive/emotional, and reward processing. This suggests that, with the aid of its widespread connectivity, the precuneus and its network can incorporate embodied and socio-cognitive/emotional aspects into mentally constructed opera scenes, required for singing. This process, which involves more than scene construction or mental imagery processing, is necessary for performing in an opera. As suggested by the network reconfiguration during an imagined singing task, we propose a novel role for the precuneus in opera performance.

## Data Availability Statement

The original contributions presented in the study are included in the article/supplementary material; further inquiries can be directed to the corresponding author/s.

## Ethics Statement

The studies involving human participants were reviewed and approved by the Ethics Committees of Sophia University and Juntendo University, Japan. The patients/participants provided their written informed consent to participate in this study.

## Author Contributions

ST and EK planned and conducted all the experiments. ST analyzed the data and wrote the manuscript. Both authors contributed to the article and approved the submitted version.

## Conflict of Interest

The authors declare that the research was conducted in the absence of any commercial or financial relationships that could be construed as a potential conflict of interest.

## Publisher’s Note

All claims expressed in this article are solely those of the authors and do not necessarily represent those of their affiliated organizations, or those of the publisher, the editors and the reviewers. Any product that may be evaluated in this article, or claim that may be made by its manufacturer, is not guaranteed or endorsed by the publisher.
